# Lipoproteins as Markers for Monitoring Cancer Progression

**DOI:** 10.1155/2021/8180424

**Published:** 2021-09-13

**Authors:** Logeswaran Maran, Auni Hamid, Shahrul Bariyah Sahul Hamid

**Affiliations:** Oncological and Radiological Sciences Cluster, Advanced Medical and Dental Institute, Universiti Sains Malaysia, Penang, Malaysia

## Abstract

Lipoproteins are among the contributors of energy for the survival of cancer cells. Studies indicate there are complex functions and metabolism of lipoproteins in cancer. The current review is aimed at providing updates from studies related to the monitoring of lipoproteins in different types of cancer. This had led to numerous clinical and experimental studies. The review covers the major lipoproteins such as LDL cholesterol (LDL-C), oxidized low-density lipoprotein cholesterol (oxLDL-C), very low-density lipoprotein cholesterol (VLDL-C), and high-density lipoprotein cholesterol (HDL-C). This is mainly due to increasing evidence from clinical and experimental studies that relate association of lipoproteins with cancer. Generally, a significant association exists between LDL-C with carcinogenesis and high oxLDL with metastasis. This warrants further investigations to include Mendelian randomization design and to be conducted in a larger population to confirm the significance of LDL-C and its oxidized form as prognostic markers of cancer.

## 1. Introduction

Cancer remains one of the major causes of mortality worldwide. The prevalence is increasing despite efforts to develop new therapeutic strategies [1]. Survival of the cancer cells can be attributed to their ability to sustain growth, evade apoptosis, invasion, metastatic characteristic, metabolic reprogramming, and undergoing hypoxia. These cells require constant supply of energy that is supplied from dietary sources. Glucose is one of the major sources of energy with the Warburg effect being well established in cancer cells. Besides glucose, cancer cells also can sustain growth from other sources of energy such as lipoproteins derived either via *de novo* synthesis or dietary intake.

The two major lipid forms that are carried by the lipoproteins are cholesterol and triglycerides [2]. Lipoproteins are mainly synthesized by the liver and intestinal cells and classified according to their density into chylomicrons (CM), very low density (VLDL-C), low-density lipoprotein (LDL-C), and high-density lipoprotein (HDL-C), while in the blood circulation, they are in a state of continuous flux due to the shifting of the composition of lipids within the lipoproteins [3]. A condition such as hypercholesterolemia is known for its role in the progression of atherosclerotic vascular disease. Abnormal lipid levels or dyslipidemia also occurs in patients with obesity and diabetes mellitus [4]. A high level of LDL-C can promote functional damage at the endothelium that leads to the development of lesions, whereas HDL-C or commonly known as good cholesterol exhibits a protective role by lowering the risk of coronary artery disease [5]. In cancer patients, a high level of HDL-C exhibits an antiproliferative effect, whereas low level of HDL-C is associated with metastasis in liver cancer patients [6]. Similarly, breast cancer patients exhibit an inverse correlation between HDL-C level and risk of cancer [7]. Dyslipidemia is highly prevalent among patients with diabetic mellitus with poorly controlled glucose level [8]. Prospective studies suggest mechanistic overlap in the pathobiology of atherosclerosis and carcinogenesis [9]. By elucidating the metabolic characteristics of lipoproteins, they can be exploited as markers for monitoring cancer progression. This review highlights the significance of LDL cholesterol (LDL-C), oxidized low-density lipoprotein cholesterol (oxLDL-C), very low-density lipoprotein cholesterol (VLDL-C), and high-density lipoprotein cholesterol (HDL-C) with carcinogenesis based on evidence gathered from clinical studies as well as experimental studies.

## 2. Methods

### 2.1. Data Sources

In the present study, the data source selection was done as recommended by the Preferred Reporting Items for Systematic Reviews and Meta-Analysis (PRISMA) guidelines [10]. The databases used to perform the literature search were Medline (PubMed), the Cochrane Central Registry of Controlled Trials, Science Direct, and Scopus articles published between 2010 and 2020. Mesh terms used as keywords included “Cancer” OR “Neoplasm” AND “Low density lipoprotein” OR “LDL-C” OR “Oxidized low density lipoprotein” OR “OxLDL-C,” “Very Low density lipoprotein” OR VLDL-C.

### 2.2. Study Selection Criteria

**Figure 1:**
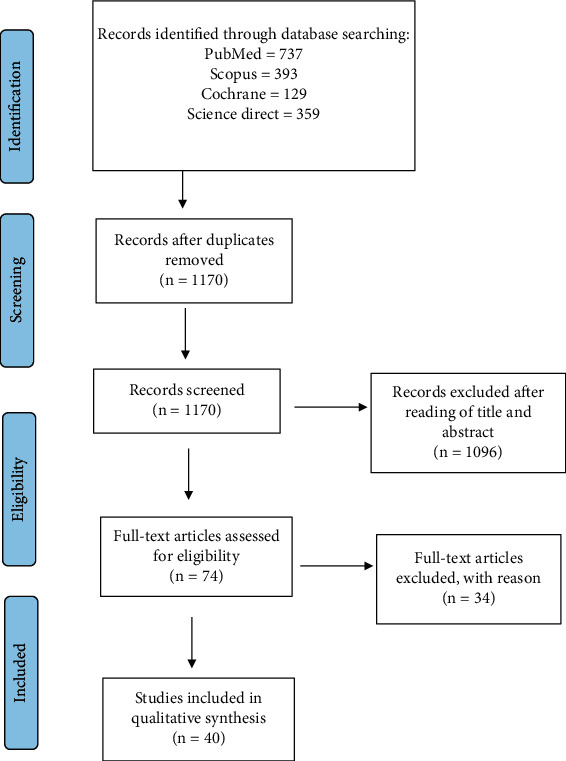
PRISMA flow diagram.

After a literature search, studies selected comprised of original articles, reviews, and clinical trials that meet the inclusion criteria: (a) cancer studies, (b) population aged ≥ 18 years, (c) studies that were published in English between 2010 and 2020, (d) completed clinical trial involving cancer patients, and (e) relevant *in vivo* and *in vitro* study. Studies excluded were those of conference proceeding, review, case report, letter to editor, protocol, and book chapter. Flow of the study selection process is summarized (Figure 1).

### 2.3. Data Collection Process and Data Items

Studies that met the inclusion criteria were chosen for evaluation by two reviewers independently. The reviewers were not blinded to each other's decision, and disagreement was resolved by the third reviewer. Later the information was retrieved from the articles using a standard data collection form. Any disagreement between individual decisions was resolved by reassessing the article. The form contains the name of the author, year, study country, study design, lipoprotein type, cancer type, sample size, and description of the main findings.

### 2.4. Risk of Bias in Individual Studies

The risk of bias is evaluated regarding the modified scale of the Agency for Healthcare Research and Quality (AHRQ) for observational studies [11]. The scale of each element is either “adequate” (A) (study meets the specific item), “inadequate” (I) (study does not meet the specific item), or “not reported” (NR) (if fail to cite a specific item).

## 3. Results

### 3.1. Clinical Studies

Database search generated 1618 records but 448 were excluded because of duplication (Figure 1). The remaining 1170 studies were screened individually based on the title and abstract. Subsequently, 1096 studies were excluded, and there were 74 full-text studies eligible for further inclusion assessment. Next, 34 full-text studies were further removed due to the exclusion criteria (Table 1). Subsequently, 21 studies that met the inclusion criteria were chosen. A summary of the characteristics of clinical studies is provided (Table 2). The risk of bias of each study is stated in Table 3.

### 3.2. Experimental Studies

The experimental studies that were selected are provided in Table 4. It includes a total 19 experimental studies that involved *in vitro* model and animal models. These studies involved various cancers like prostate cancer, breast cancer, gastric cancer, B cell tumor, chronic lymphocytic leukemia, liver cancer, hepatocellular carcinoma, ovarian cancer, and lung adenocarcinoma.

## 4. Discussion

In this review, a total of 21 clinical studies involve case control observational, Mendelian randomization, prospective, and retrospective studies. Jamnagerwalla et al. tested the association between serum total cholesterol, LDL-C, HDL-C, and prostate cancer risk. A post hoc analysis was performed on data obtained from 4974 nonstatin users with elevated prostate specific antigen and a negative baseline biopsy [12]. They found that high total cholesterol and high HDL-C were significantly associated with an increased risk of high-grade prostate cancer.

Yang et al. investigated the association between oxidized low-density lipoprotein with hematological malignancies [13]. A total of 39 patients with leukemia and 19 without malignancies were recruited to assess the level of oxidized low-density lipoprotein. Findings suggest that the patients with leukemia had significantly higher oxidized low-density lipoprotein than those without cancer. However, the study limitation was the small sample size.

Diakowska et al. assessed the level of oxidized low-density lipoprotein in 73 colorectal carcinoma (CRC) patients and 30 healthy control participants [14]. Body mass index (BMI) was matched in both groups despite the mean age difference. The parameters measured included total cholesterol, HDL-C, LDL-C, triglycerides, and glucose. There was an insignificant difference in serum oxidized low-density lipoprotein between colorectal cancer patients and the healthy group. However, the level of oxidized low-density lipoprotein was significantly higher in patients with early stage of primary tumor than patients with advanced stage. Similar to Yang et al. [13], the study had limited sample size. Besides, there was also lack of information on statin use.

Hu et al. [15] studied the prediction capability of lipid derivatives in the prognosis of postoperative gastric cancer. The lipid derivatives are better predictors than single lipid parameter [16]. Therefore. they compared three lipid derivatives that comprised of the ratio of total cholesterol minus HDL-C to HDL-C termed as an atherogenic index (AI), the ratio of triglycerides to HDL-C (THR), and the ratio of LDL-C to HDL-C referred to as LHR [15]. The study enrolled 3012 gastric cancer patients with a 5-year follow-up duration. Findings gathered indicate that AI and LHR have the prediction capability of gastric cancer mortality particularly in male patients or TNM stages I and II or intestinal-type gastric cancer patients with normal BMI and without hypertension. Furthermore, the study indicated that the patients with a lower level of AI, LHR, and THR had better survival rates than those with high values. The authors also recommended for the clinical management to incorporate monitoring of HDL-C to improve the survival of patients with gastric cancer. The study involved data collection from a single center, and another limitation was the lack of information on the use of lipid-lowering drugs.

Bhat et al. studied the association of lipids and body mass index (BMI) with risk of breast cancer [17]. The study involved 60 breast cancer patients and 60 healthy females as controls of similar age. They reported that the breast cancer patients had slightly higher BMI than the control. Almost 16% of the premenopausal women exhibited high total cholesterol than were the healthy controls, whereas postmenopausal patients had 22% higher triglyceride level than healthy controls. However, no significant difference was observed in HDL-C level between the breast cancer patients and controls. Comparison with LDL-C cholesterol level indicated that it was 22% higher in premenopausal patients and 12% in the postmenopausal patients than the healthy controls. The study indicates that an increase in BMI and obesity was associated with the possible risk of breast cancer. However, the effects of other confounding factors were not included in the analysis.

In another study, Llanos et al. described the possible association between lipoprotein levels and breast cancer risk [18]. The study involved 97 breast cancer patients and 102 control with a mean age of 57 years. Findings suggest that total cholesterol and LDL-C levels were lower in patients than in the control group. It also found a significant risk of breast cancer associated with low HDL-C (OR = 1.99) but the low risk with high LDL-C (OR = 0.41). They found a significant reduction in breast cancer risk with high total cholesterol (OR = 0.46). However, the study had a lack of information in regard to the menopausal status, statin use, and small sample size.

Benn et al. performed Mendelian randomization analysis to examine the potential causality of genotypes that are associated with a decreased level of LDL-C and risk of cancer [19]. A Mendelian randomization is an epidemiological approach for studying potential causal relationship as it takes into consideration of the confounding factors. The population studied were those from the Copenhagen City Heart Study (CCHS) and Copenhagen General Population Study (CGPS). Information on genotype was available for 10,293 (CCHS) and 56,624 (CGPS) patients with invasion. The study involved 15-year follow-up duration. A sequence detection system was used to identify genotypes encoding for single-nucleotide polymorphism of *PCSK9*, *ABCC8*, and *APOE* genes in 11,110 patients. Analysis done included multifactorial adjustment of age, sex, BMI, hypertension, diabetes mellitus, smoking, and statin use. Interestingly, findings suggest that low LDL-C and the genotypes studied were not associated with an increased risk of cancer. The authors concluded that low LDL-C is secondary to preclinical cancer that was probably due to cholesterol absorption, transport, metabolism, or utilization. However, these findings were limited to three genotypes and also the Caucasian population. It may not be applicable to other ethnic groups. In another report, Nowak and Ärnlöv performed a two-sample Mendelian randomization analysis on data retrieved from more than 400,000 patients [20]. This was done to identify the causal relationship between LDL-C, HDL-C, triglycerides, and variants of genes with risk of developing either estrogen receptor-positive or estrogen receptor-negative breast cancer. Data analyzed were from the Global Lipid Genetics Consortium (GLGC) and the Breast Cancer Association Consortium (BCAC) that had more than 180,000 patients in each Consortium. In contrast to a previous report by Benn et al., this study indicated the presence of an association between genetically raised LDL-C with increased risk of breast cancer mainly in patients with estrogen receptor positive. Their finding suggests that low LDL-C variant (PCSK9) provides a protective effect. On the other hand, HDL-C was not associated with breast cancer. Similarly, triglycerides were not associated with increased risk of breast cancer independently. However, their study had a lack of information on the menopausal status. It is known that endocrine changes that occur in menopause can modulate plasma lipid parameters. Therefore, the authors recommended further investigation on genetic and drug exposure to address the effects of statin on breast cancer risk.

A retrospective cohort analysis was done previously on patients with primary ductal carcinoma *in situ* and invasive breast cancer (Kaiser Permanente Northern California Registry), where they were given lipophilic statins (*n* = 2830) [21]. The results indicated statin use for more than a year prior the diagnosis significantly decreased the proportion of ER/PR-negative patients than those without any statin use or with less than a year of statin use. This suggests that statin can significantly be associated with low-grade tumors and *in situ* localization. Statin may exert its effect by modifying the phenotype of breast cancer rather than reducing the total number of breast cancer cases. Despite these findings, the study did not assess the overall breast cancer risk reduction by lipophilic statins.

In 2013, a study by Laisupasin et al. involved patients with early breast cancer that was limited to breast and regional lymph nodes (*n* = 249) and normal participants (*n* = 154) [22]. According to their study, triglyceride, LDL-C, and VLDL-C were significantly higher in breast cancer patients than those without cancer. However, there were HDL-C and total cholesterol did not differ between cancer and normal participants. Among the limitation of their study was the lack of information on stages of cancer, types of cancer, statin use, and menopausal status. These parameters are known to regulate lipoprotein levels.

Later in 2015, Wan et al. performed a retrospective study to investigate the effects of cardiovascular markers. They monitored the changes of oxidized LDL-C, apolipoprotein B100, and apolipoprotein B48 in those with localized or lymph node metastatic prostate cancer [23]. The study involved 50 patients and 25 healthy individuals as the control participants. In this study, more than 64% of the patients were overweight. The findings indicated significantly higher oxidized LDL-C in primary metastatic prostate cancer than those with benign prostate cancer hypertrophy. The authors recommended extended lymph node dissection and closer examination of surgical margin particularly in patients with high oxidized LDL-C. Gene association analysis was done on 256 prostate cancer (PCa) patients for oxidized low-density lipoprotein (OLR1) receptors. The study suggests that the probability of positive lymph nodes increased significantly when expression of OLR1 was high, Gleason scores more than 7, and high prostate specific antigen. This indicates that the oxidized LDL-C is a pivotal factor for the progression of PCa and a suitable as a prognostic marker. According to the authors, the study might have selection bias because it was restricted to a single center. They recommended further studies should involve multicenter and multiethnicity. These approaches will render a better understanding of the association of oxLDL-C with prostate cancer risk.

Raza et al. conducted a cross-sectional study among 208 breast cancer patients who had infiltrating ductal carcinoma without treatment and 176 matched control subjects [24]. The findings indicate significantly higher glucose, total cholesterol, triglycerides, and LDL-C in patients than control subjects except for HDL-C. Furthermore, patients with lymph node metastasis in all tumor grades had significantly higher glucose and hyperlipidemia than patients without metastasis. The variation in lipid profile and glucose was higher in patients with tumors size of 2.5 cm than those with tumors size of 5 cm. The changes in the lipoproteins can be attributed to the underlying metabolism due to tumor activity. These findings also suggest that there was no independent relation between hyperlipidemia and disease-free survival. The study was restricted to a single center and did not provide information in regard to the menopausal status of the participants.

Rodrigues dos Santos et al. conducted a prospective study to assess the lipid profiles in a cohort of 244 patients who had invasive breast cancer [25, 26]. The analysis was performed by taking into consideration of the important confounding factors such as menopause, BMI, age, family history, antidiabetic drug and lipid-lowering drug intake. Results of hazard ratio were 2.4, 1.9, and 1.88 for LDL-C, total cholesterol, and triglycerides, respectively. Further univariate analysis indicated that all the parameters were significantly different. The study reported that the LDL-C level of more than 117 mg/dL is a predictive factor of tumor stage at diagnosis. After 25 months of follow-up, disease-free survival was significantly reduced in patients with >144 mg/dL LDL-C than those with <117 mg/dL (88.3% vs. 100%). Moreover, the mean BMI among those with >144 mg/dL LDL-C was 27 kg/m^2^ compared to 25 kg/m^2^ in the other group. LDL-C was significantly associated with breast cancer progression and can be used in the identification and follow-up of high-risk patients. On the other hand, high HDL-C was associated with low risk of premenopausal breast cancer. According to the study, high HDL − C > 60 mg/dL can decrease the risk by 0.49-fold than <50 mg/dL HDL-C. This study performed the analysis by including most of the confounding factors except for smoking and diet.

Xie and Shao performed a retrospective study on 1140 patients with nasopharyngeal carcinoma (NPC) [27]. These patients were grouped into those with eye metastasis and without eye metastasis. The purpose of the study was to identify potential risk factors with clinical significance for the monitoring of NPC. The study involved monitoring of total cholesterol, triglycerides, HDL-C, LDL-C, Apo A1, and Apo B. There was a significant reduction of triglycerides and total cholesterol in NPC patients with eye metastasis. Findings suggest that the total cholesterol and triglycerides can be risk factors of eye metastasis particularly in male NPC patients. The authors recommended further investigation to monitor triglycerides in different types of cancers. However, the study also had limitations of small sample size in the eye metastasis patients and restricted to a province. Therefore, it may not be applicable to the general NPC population. Furthermore, there was lack of information regarding BMI, weight, glucose, statin use, or medications taken. Future studies must design analysis that incorporates all the confounding factors to verify these findings.

Ibaraki Prefectural Health Study (2013) was aimed at examining the relationship between LDL-C level and liver cancer mortality [28]. It is a community-based large cohort study that involved 16,217 liver cancer patients with s follow-up duration of 14 years. The study concluded that the mortality from liver cancer was highest in the group with the lowest LDL-C level. Furthermore, hazard ratios for liver cirrhosis were higher in patients with less than 80 mg/dL LDL-C than those with more than 80 mg/dL. The possible mechanism of the association between total cholesterol and liver cancer can be attributed to hepatic dysfunction or chronic inflammatory change that acts as a promoter in the multistep process of carcinogenesis. However, the study did not determine whether patients had a history of viral hepatitis or alcohol hepatitis. Interestingly, the cholesterol level was higher than fasting participants and may contribute to selection bias.

Ma et al. reported that oxLDL was positively correlated with lymphatic metastasis in 28 gastric cancer patients [29]. They concluded that the reduction of oxLDL could be an approach for the prevention and intervention of early lymph node metastasis in gastric cancer patients. According to the authors, a larger population-based study that considers factors such as age, sex, and BMI is necessary to confirm the findings.

Crespo-Sanjuan et al. performed an observational study with a 3-year follow-up involving 128 patients with colorectal carcinoma. The study reported elevation of oxLDL starting at the polyp stage in patients without dysplasia and suggests the relevance of oxLDL as an early marker of cancer risk. Comparison with HDL-C was done, and results indicated that HDL-C was low in these patients indicating its protective function [30]. However, the authors did not state their limitations.

According to Lofterød et al., findings from a population-based survival study of 464 breast cancer patients indicated a significant interaction between triglycerides and triple negative breast cancer (TNBC) and HDL-C to total cholesterol ratio with TNBC. The 5-year overall survival was 19% low patients with high triglycerides. TNBC patients with the highest triglycerides had 24% lower 5-year breast cancer-free survival than those having the lowest triglycerides. TNBC is associated with poor prognosis, and identification of clinically available markers is important to improve the outcomes for this subgroup of patients [31]. Similarly, HDL-C to total cholesterol ratio was inversely correlated with overall mortality in TNBC patients. The analysis done included BMI, glucose, blood pressure, physical activity, alcohol, and smoking habits. The authors also performed an adjustment on time since the last meal since the samples were collected in nonfasting state. The study recommends for larger sample size in each molecular subclass in future study designs.

Yuan et al. evaluated the prognostic significance of preoperative serum lipids in 99 patients with gallbladder cancer [32]. They included confounding factors such as gender, age, tumor size, lymph nodes, and TNM stage in the analysis. Findings suggest that low HDL-C level was associated with poor survival after surgery. The authors proposed the combination of HDL-C and lymph node metastasis as predictor of prognosis in gallbladder cancer. The limitation of this study was the selection of patients undergoing surgery only and the small sample size.

A recent prospective cohort study by Brantley et al. [33] involved 341 CRC patients, and their lipoprotein changes were monitored for a year. Analysis performed considered factors such as age, sex, year of diagnosis, chemotherapy treatment stage, NSAID, aspirin, and statin use. The findings indicated that high HDL-C has a beneficial effect on recurrence-free survival particularly in patients given statins. Nevertheless, an increase of LDL-C and triglycerides was not associated with CRC recurrence. This study was done in a small cohort with a limited number of recurrences.

In another study by Mosapour et al., 35 women with benign fibroadenoma and 50 ductal cell carcinomas were recruited [34]. The study evaluated the potential association between serum VLDL-C and correlation with the clinicopathological features. According to the study, VLDL-C was significantly lower in patients with breast carcinoma and benign tumor than normal participants. Interestingly, tissue VLDL-C was high in premenopausal breast cancer patients. A high level of triglycerides can lead to a low level of sex hormone-binding globulin and lead to an increase in free estradiol concentration. Consequently, these physiological changes may increase the risk of breast cancer. However, there was no significant association between tissue VLDL-C and prognostic factors. This study did not include the confounding factors in the analysis.

Brantley et al. conducted a study on colorectal cancer patients to monitor changes in lipoproteins. The study found an increase of HDL-C had a beneficial effect on recurrence-free survival particularly in statin users. However, the study demonstrated there was no association with colorectal cancer recurrence with an increase in LDL-C and triglycerides [33].

Taken together, high total cholesterol and high HDL-C were associated with prostate cancer [12]. Similarly, higher LDL-C was associated with metastasis [24] and predictive risk in breast cancer [17, 19–22, 25, 26] and gastric cancer [15]. In contrast, evidence from Mendelian randomization analysis that excluded secondary hyperlipidemia found possible relation of low LDL-C as a consequence of preclinical cancers [19]. However, the study did not include other types of lipoproteins to compare the differences. Low LDL-C has also been associated with liver cancer [28], and a significant reduction in total cholesterol occurred among male patients with NPC [27]. However, larger population studies are needed to further confirm these findings. Besides native LDL-C, recent findings suggest a possible causative role of oxLDL-C in cancer [13, 14, 23, 29, 30].

High HDL-C level has been linked with significant risk reduction mainly in postmenopausal than premenopausal breast cancer patients. Hormonal changes can influence the relationship between HDL-C and the risk of breast cancer [17, 21]. This was in-line with previous finding from gastric cancer, TNBC, gallbladder, and CRC patients who exhibited improved survival with high HDL-C [15, 30–33]. In contrast, high HDL-C was significantly associated with stages of lymph node metastasis and can be a prognostic marker [22, 24]. However, confounding factors such as BMI, statin use, smoking, diet, weight loss, total fat loss, compliance to exercise, and expert supervision were not controlled in both of these studies. These factors are known to influence lipoprotein levels in blood circulation. Besides, different treatments were given for the management of the high levels of LDL-C in the patients. Therefore, the results can vary due to these factors, whereas the relation between VLDL-C and triglycerides with breast cancer risk was reported previously [17, 21, 22, 31, 34] and NPC [27].

Reprogramming of lipid metabolism occurs in carcinogenesis. This is supported by evidence from the *in vivo* and *in vitro* studies that reveal the underlying molecular mechanisms. Lu et al. reported LDL-C and triglycerides were able to promote breast cancer cell aggressiveness [35]. The significance of cholesterol was demonstrated by a hypercholesterolemic mouse model study, where cholesterol had induced larger and more proliferative breast cancer cell along with the occurrence of lung metastases [36].

TNBC cell lines (MDA-MB-231 and MDA-MB-436) can accumulate more lipid droplets than in the estrogen receptor-positive cell line (MCF7). Sobot et al. found high expression of LDL receptor in the TNBC cells, indicating high demand for LDL-C [37]. It corresponds to greater activity of LDL uptake, acyl-CoA: cholesterol acyltransferase 1 (ACAT1), and low cholesterol synthesis by the estrogen receptor-negative breast cancer cells [38]. According to Zhao et al., spindlin 1 (SPIN1) is involved in the organization of spindle and the stability of chromosome that plays an important role in carcinogenesis. SPIN1 modulates abnormal lipid metabolism by increasing intracellular cholesterol, triglycerides, and lipid droplets in hepatoma cells. Analysis of liver cancer tissue samples provided evidence of higher expression of SPIN1 than peritumor tissue samples. A similar result was obtained using the hepatoma cell line, where there was upregulation of FASN. The SPIN1 stimulates the growth of liver cancer via the SREBP1c-triggered FASN signaling pathway. Therefore, it may serve as a novel target for hepatocellular carcinoma [39]. In the immune response, V*γ*9V*δ*2 T cells display antitumor functions through cytotoxicity and interferon gamma (IFN*γ*) production upon activation. However, uptake of LDL-C led to impaired IFN*γ* production and reduced breast cancer cell death (Rodrigues et al., 2018). These findings suggest that LDL-C can be a key player in sustaining the survival of cancer cells. In another study by Murtola et al., LDL-C was able to stimulate the growth of prostate cancer cells [40]. Besides that, cancer cells can survive due to the *de novo* synthesis of lipids occur with an increase in the expression of the biosynthetic machinery without cholesterol efflux. This suggests the occurrence of reprogramming of cholesterol metabolism in the cancer cells. Rodrigues dos Santos et al. also provided evidence with regard to the involvement of LDL-C in cancer cell proliferation [25, 26]. The downstream signaling is dependent on the activation of Akt and ERK pathways. LDL-C causes low expression of adhesion molecules such as cadherin-related family member 3, CD226, Claudin 7, and Ocludin genes. The degradation of LDL-C generates products that can promote survival of the proliferating chronic lymphocytic leukemia (CLL) cells and cause an increase of plasma membrane cholesterol signaling molecules like STAT3 tyrosine-phosphorylated and activated CLL cell number [41]. However, the signaling effects of LDL-C do not occur in normal lymphocytes. Staphylococcal nuclease domain containing protein 1 (SND1) is an oncogenic protein involved in lipid metabolism. Its overexpression induces cholesterogenesis and cholesterol ester accumulation. Apart from that, it causes lower use of fatty acid for triglyceride synthesis than cholesterol synthesis, therefore leading to lower triglyceride concentration [42]. Most of the experimental studies indicated significant involvement of LDL-C in carcinogenesis. Hence, recent therapeutic strategies use LDL as a drug carrier. LDL nanoparticles have been used as anticancer therapeutic, for example, the formulation of docosahexanoic acid (DHA) into LDL. It can cause selective cytotoxicity to hepatocellular carcinoma cells [43]. Cancer cells are known to upregulate the generation of reactive oxygen species (ROS). Therefore, the higher concentration of ROS is present in cancer cells than in normal cells. This leads to enhanced metabolism of DHA to generate oxidized products that lead to protein dysfunction and selective toxicity against cancer cells. DHA also causes mitochondrial and nuclear damage to murine liver cancer cell [44]. LDL-DHA is reported to act on hepatocellular carcinoma cells via the ferroptosis pathway [45].

*In vitro* and *in vivo* studies demonstrate that LDL-C is increasingly associated with carcinogenesis. This has been attributed to an increase in LDL-C uptake and receptor activity [46–52]. Additionally, oxidative stress can induce carcinogenesis and oxidation of low-density lipoprotein. The experimental studies suggest that the oxidized LDL may be an independent mitogenic factor [53]. Oxidized LDL-C (oxLDL) can stimulate the production of ROS (C.-S. [54]). As a consequence, high ROS will then activate intracellular signaling through tyrosine kinase, phosphatidylinositide 3-kinase (PI3K)/Akt, mitogen-activated protein kinases (MAPK), and nuclear factor-kappaB (NF*κ*B) [55, 56]. A previous study in ovarian cancer cells indicates that low oxLDL was sufficient to stimulate the proliferation of cells than native LDL-C and caused low chemosensitivity in the ovarian carcinoma cells [57]. In the MMTV-PyMT Tg mouse model, oxLDL induced growth of tumor but intake of phytosterol-enriched diet prevented LDL from oxidation [58]. Wan et al. reported that oxLDL-C caused cancer cell proliferation by targeting the cell cycle phases [23]. According to González-Chavarría et al., oxLDL can activate LOX1 in prostate cancer cells, by which led to epithelial-mesenchymal transition (EMT) characteristics. This mechanism further stimulates the production of mesenchymal markers and EMT-associated transcription factors (snail and slug) [59]. EMT has been associated with initial stages of invasion and migration that are essential for metastasis. Wang et al. demonstrated that C/EBP6 was able to recruit oncogene NCOA3 that consequently transcriptionally activates slug. It is a canonical EMT transcription factor that stimulates the uptake of oxLDL to promote metastasis [60]. According to Khaidakov and Mehta, LDL-C can activate NADPH oxidase and increase the production of superoxide [61]. Furthermore, oxLDL stimulates has-miR-21 that is recognized for its association with the pathobiology of cancer. The study reports on the roles of oxLDL in the activation of the PI3K/Akt pathway. In another study by Lu et al., there was an increase in the viability of MCF7 cells (ER+, PR+, and HER2-) after exposure to VLDL-C than HDL-C [35], Similarly, TNBC cell (MDA-MB-231) also exhibited a high percentage of cell viability when supplemented with the artherogenic-subfraction of LDL-C and VLDL-C. Huang et al. performed a study on the syngeneic tumor graft model to assess whether the development of tumor may regulate the host lipid metabolism. They reported that the synthesis of VLDL was induced by B cell tumor, and the tumor-mediated hyperlipidemia supplies LDL-C for the growth of tumor cells (J. [62]). According to a previous report, triglyceride-rich VLDL is the precursor of LDL that is catabolized slowly [63, 64]. This is also supported by recent studies that associated VLDL with breast cancer [34, 65].

The limitation of the review is that some of the studies did not eliminate confounding factors in the analyses. However, the search protocol was done according to PRISMA guidelines, and the source of literature is available in the databases listed.

## 5. Conclusion

These findings suggest that LDL-C could act as a marker for monitoring the progression of various cancers. Early monitoring would be able to stratify patients into low- and high-risk group according to their LDL-C level. Besides, emerging reports indicate the involvement of oxLDL in metastasis. Therefore, this warrants future studies with a larger sample size with Mendelian randomization design to confirm the underlying role of lipoproteins as prognostic markers.

## Figures and Tables

**Table 1:** Full-text articles that were excluded.

Articles excluded	Reason for exclusion	Number of articles
Alves Dias J., 2016 Chen KC. et al. 2015 Cheng D. et al. 2016 Crespo-Sanjuán J. et al. 2015 Gao H., 2013 Lu H. et al. 2015 Morel S. et al. 2017 Pan B. et al. 2012 Sabnis N. et al. 2012 Ruscica M. et al. 2018 Tamura T. et al. 2012 Wu J. et al. 2018 Yao JJ. et al. 2018 Zhang F. et al. 2016	Letter/correspondence/commentary/response/communication/editorials/	14

Abu Saadeh F. et al. 2013 Eliaz I. et al. 2016 Henrich SE. et al. 2019 Hescot S. et al. 2015 Kanzaki M. et al. 2012 Li X. et al. 2017 Martin LJ. et al. 2015 Pace G. et al. 2010 Sonowal R. et al. 2019 Sun P. et al. 2017 Tsouma I. et al. 2014 Wolny-Rokicka. et al. 2017	Practice guidelines/pilot studies	12

Danilo C. et al. 2013 Flote VG. et al. 2016 Gupta RK. et al. 2012 Herrera LV. et al. 2018 Li M., 2018 Keller J. et al. 2015 Pires LA. et al. 2012 Velagapudi S. et al. 2018 Wolfe AR. et al. 2016	Case report	9

Albers JJ. et al. 2012 Chen P. et al. 2017 Fagan-Solis KD. et al. 2014 Grosman H. et al. 2010 Mahmoudian M. et al. 2018 Podzielinski I. et al. 2013 Youn Nam S. et al. 2019	Study protocols	7

Bérard E. et al. 2011 Chen CH. et al. 2019 Hlebowicz J. et al. 2011 Indu MS. et al. 2018 Katzke VA. et al. 2017 Kumar P. et al. 2012 Madssen TS. et al. 2018 Notarnicola M. 2019 Pandeya DR., 2018 Reynolds L. et al. 2014 Sigal GA. et al. 2019 Song J. et al. 2019 Tan MC. et al. 2019 Patel R. et al. 2017 Ye J. et al. 2018 Zhu C. et al. 2017	Unrelated to lipoprotein studies, failure to provide clinical information or failure to present data clearly	16

**Table 2:** Summary and characteristics of the clinical studies.

Author/year	Country	Study design	Type of lipoprotein	Sample	Type of cancer	Main findings
Brantley et al. [33] Ref 32	USA	Prospective cohort study	TG, LDL-C, HDL-C	Case: 341	Colorectal cancer	Increase HDL-C exhibits beneficial effect on recurrence-free survival among statin users. Serum LDL-C and triglycerides were not associated with colorectal cancer recurrence.
Benn et al. [19] Ref 18	Denmark	Mendelian randomization study	LDL-C	Case: 70,179	Invasive cancer	LDL-C level was associated with an increased cancer risk 43% increase (95%confidence interval [CI] = 15% to 79% increase).
Bhat et al. [17] Ref 16	India	Case-control study	TC, TG, HDL-C, and LDL-C	Total: 120 Case: 60 Control: 60	Breast cancer	LDL-C increased significantly (*r*^2^ = 0.031; *p* = 0.0003) only in the postmenopausal women than controls but HDL-C remains unchanged.
Crespo-Sanjuan et al. [30] Ref 28	Spain	Case-control study	oxLDL, HDL-C	Total: 142 Case: 128 Control: 14	Colorectal cancer	Increase of oxLDL at the polyp stage in patients without dysplasia indicates relevance of oxLDL as an early risk marker. Increase of HDL-C in patients shows that HDL-C has protective effect.
Diakowska et al. [14] Ref 13	Poland	Case-control observational study	TC, TG, HDL-C, LDL-C	Total: 108 Case: 73 Control: 35	Colorectal cancer	oxLDL was significantly higher in early stage of primary tumor than advanced stage tumor.
Hu et al. [15] Ref 15	China	FIESTA observational study	TC, TG, HDL-C, LDL-C	Case: 3012	Gastric cancer	Atherogenic index and LHR are better predictor of gastric cancer mortality in male patients or TNM stages I and II or intestinal type gastric cancer.
Jamnagerwalla et al. [12] Ref 11	USA	REDUCE observational study	TC, HDL-C, LDL-C	Case: 4974	Prostate cancer	High total cholesterol and high HDL-C were associated with increased risk of high-grade prostate cancer.
Kumar et al. [21] Ref 20	India	Case-control study	TC, TG, HDL-C, LDL-C, and VLDL-C	Total: 200 Case: 100 Control: 100	Breast cancer	LDL-C, TG, and TC were associated with breast cancer (*p* < 0.05). However, there was no association with different grades of carcinoma. LDL-C, TG, and TC may have some role in aetiology of breast cancer.
Laisupasin et al. [22] Ref 21	Thailand	Case-control study	TC, TG, HDL-C, LDL-C, and VLDL-C	Total: 403 Case: 249 Control: 154	Breast cancer	TG, LDL-C, and VLDL-C levels in breast cancer group were significantly increased than normal control group (*p* < 0.001). HDL-C and TC levels were not associated.
Llanos et al. [18] Ref 17	USA	Case-control study	TC, TG, HDL-C, and LDL-C	Total: 199 Case: 97 Control: 102	Breast cancer	Increase of LDL-C was inversely associated with breast cancer risk (OR = 0.41 (0.21–0.81)). Low level of HDL-C was associated with significant increase in breast cancer risk (OR = 1.99 (1.06–3.74)).
Lofterød et al. [31] Ref 29	Norway	Prospective study Tromsø study	Total cholesterol Triglycerides HDL-C	Case: 464	Breast cancer	Significant interaction between triglycerides and TNBC and HDL-C to total cholesterol ratio and TNBC Strong association between prediagnostic triglycerides and overall mortality and breast cancer-free interval in TNBC patients 5-year overall survival lower in TNBC patients having high triglycerides HER2 patients with high triglycerides and HDL-C to total cholesterol ratio exhibit inverse association with overall mortality.
Ma et al. [29] Ref 27	China	Observational study	oxLDL	Case: 28	Gastric cancer	Concentration of plasma oxLDL is significantly increased at higher stage of lymph node metastasis.
Mosapour et al. [34] Ref 31	Iran	Case-control study	VLDL-C	Total: 105 Case: malignant (50), benign (35) Control: 20	Breast cancer	Positive association between VLDL-C with grade, Ki67, and size of tumor High level of VLDL-C in premenopausal patients
Nowak et al. [20] Ref 19	Sweden	Mendelian randomization study	HDL-C and LDL-C	>400,000	Breast cancer	Raised LDL-C increased the risk of breast cancer (OR = 1.09 (1.02–1.18)) and ER-positive breast cancer (OR = 1.14 (1.05–1.24)). Raised HDL-C increased the risk of ER-positive breast cancer (OR = 1.13 (1.01–1.26)).
Raza et al. [24] Ref 23	Pakistan	Case-control study	TC, TG, HDL-C, and LDL-C	Total: 384 Case: 208 Control: 176	Breast cancer	Increase in TC (4%), LDL-C (23%), and TG (11%) was observed from tumor grades I and II, significantly high (*p* < 0.05) compared to control subjects.
Rodrigues dos Santos et al. [25, 26] Ref 24	Portugal	Prospective	TC, TG, HDL-C, and LDL-C	244	Breast cancer	Systemic level of LDL-C was correlated positively with tumor size (Spearman's *r* = 0.199, *p* = 0.002).
Saito et al. [28] Ref 26	Japan	Prospective	LDL-C	16,217	Liver cancer	Low LDL-C 4.33 (95% confidence interval [CI]: 1.94, 9.68) was associated with elevated risk of mortality in liver cancer.
Wan et al. [23] Ref 22	China	Case-control study	oxLDL-C	Total: 100 Case: 75 Control: 25	Prostate cancer	oxLDL-C level was significantly correlated with N stage of prostate cancer.
Xie et al. [27] Ref 25	China	Case-control study	TC, TG, HDL-C, and LDL-C	Total: 1140 Case: 16 Control: 1124	Nasopharyngeal carcinoma	TG and TC were independent risk factors in male NPC patients with *p* = 0.004 and *p* < 0.001, respectively.
Yang et al. [13] Ref 12	China	Case-control study	TC, TG, HDL-C, LDL-C, oxLDL-C	Total: 67 Case: 39 Control: 19 Healthy: 9	Leukemia	oxLDL was significantly higher in patients with leukemia than those without malignancies.
Yuan et al. [32] Ref 30	China	Retrospective study	TC, TG, HDL-C, LDL-C, Apo A, Apo B	Case: 99	Gallbladder cancer	Decrease in HDL-C correlated with poor survival

^∗^TC: total cholesterol; TG: triglycerides; HDL-C: high-density lipoprotein cholesterol; LDL-C: low-density lipoprotein cholesterol; VLDL-C: very low-density lipoprotein cholesterol; oxLDL-C: oxidized low-density lipoprotein cholesterol.

**Table 3:** List of the risk of bias in the studies.

Domain	Elements	Benn et al. [19]	Bhat et al. [17]	Brantley et al. [33]	Crespo et al. [30]	Diakowska et al. [14]	Hu et al. [15]	Jamnagerwalla et al. [12]	Kumar et al. [21]	Laisupasin et al. [22]	Lofterød et al. [31]	Llanos et al. [18]	Ma et al. [29]	Mosapasour et al. [34]	Nowak et al. [20]	Raza et al. [24]	Rodrigues dos Santos et al. [25, 26]	Saito et al. [28]	Wan et al. [23]	Xie et al. [27]	Yang et al. [13]	Yuan et al. [32]
Study question	Clearly focused and appropriate question	A	A	A	A	A	A	A	A	A	A	A	A	A	A	A	A	A	A	A	A	A
Study population	Description of study population	A	A	A	A	A	A	A	A	A	A	A	A	A	A	A	A	A	A	A	A	A
	Sample size justification	A	A	A	A	A	A	A	A	A	A	A	NR	NR	A	A	A	A	A	A	A	A
Comparability of subjects	Specific inclusion/exclusion criteria	A	A	A	A	A	A	A	A	A	A	A	A	A	A	A	A	A	A	A	A	A
	Criteria applied equally to all groups	A	A	A	A	A	A	A	A	A	A	A	A	A	A	A	A	A	A	A	A	A
	Comparability of groups at baseline with regard to disease status and prognostic factors	A	a	A	A	a	A	A	A	a	a	a	a	A	a	a	A	A	A	a	A	a
	Study groups comparable to nonparticipants with regard to confounding factors	a	A	a	a	A	a	a	a	A	a	a	a	I	a	I	a	a	NR	a	NR	a
	Use of concurrent controls	A	A	a	a	A	a	a	a	A	a	a	a	A	a	A	a	a	A	a	I	a
	Comparability of follow-up among groups	A	a	a	A	a	A	A	a	a	a	a	a	a	a	a	A	A	a	a	a	a
Exposure or intervention	Clear definition of exposure	A	A	A	A	A	A	A	A	A	A	A	A	A	A	A	A	A	A	A	A	A
	Measurement method standard, valid, and reliable	A	A	A	A	A	A	A	A	A	A	A	A	A	A	A	A	A	A	A	A	A
	Exposure measured equally in all study groups	A	A	A	A	A	A	A	A	A	A	A	A	A	A	A	A	A	A	A	A	A
Outcome measurement	Primary/secondary outcomes clearly defined	A	A	A	A	A	A	A	A	A	A	A	A	A	A	A	A	A	A	A	A	A
	Outcomes assessed blind to exposure or intervention status	a	a	a	a	a	a	A	a	a	a	a	a	a	a	a	a	a	a	a	a	A
	Method of outcome assessment standard, valid, and reliable	A	A	A	A	A	A	A	A	A	A	A	A	A	A	A	A	A	A	A	A	A
	Length of follow-up adequate for question	A	a	A	A	a	A	A	a	a	a	a	a	a	a	a	A	A	a	a	a	a
Statistical analysis	Statistical tests appropriate	A	A	A	A	A	A	A	A	A	A	A	A	I	A	A	A	A	A	A	A	A
	Multiple comparisons taken into consideration	A	NR	A	A	I	A	A	A	NR	A	NR	NR	NR	A	NR	A	A	A	A	NR	A
	Modelling and multivariate techniques appropriate	A	NR	A	A	A	A	A	A	NR	A	NR	NR	NR	A	NR	A	A	A	A	NR	A
	Power calculation provided	A	NR	NR	NR	A	A	A	A	NR	A	A	NR	NR	A	NR	NR	NR	NR	NR	NR	NR
	Assessment of confounding variables	A	NR	A	A	A	A	A	A	NR	A	a	NR	NR	A	NR	A	A	NR	NR	NR	A
	Dose–response assessment, if appropriate	a	a	a	a	a	a	a	a	a	a	a	a	a	a	a	a	a	a	a	a	a
Results	Measure of effect for outcomes and appropriate measure of precision	A	A	A	A	A	A	A	A	A	A	A	A	A	A	A	A	A	A	A	A	A
	Adequacy of follow-up for each study group	A	a	A	A	a	A	A	a	a	a	a	a	a	a	a	A	A	a	a	a	a
Discussion	Conclusions supported by results with biases and limitations taken into consideration	A	a	A	NR	A	A	A	A	A	A	A	NR	I	A	I	A	A	A	A	I	I
Funding or sponsorship	Type and sources of support for study	A	NR	A	A	NR	A	A	NR	A	A	A	A	A	A	NR	A	A	NR	A	A	A

A: adequate; I: inadequate; NR: not reported; a: not applicable to the study design.

**Table 4:** Summary and characteristics of the experimental studies.

Author/year	Country	Study design	Type of lipoprotein	Sample	Type of cancer	Main findings
González-Chavarría et al. [59] Ref 58	Chile	In vitro	oxLDL	Human prostate cancer cells (C4-2, C4-2B,: NCAP, PC3, DU-145)	Prostate cancer	oxLDL induces EMT characteristics.
Huang et al. [62] Ref 61	Austria	In vitro In vivo	Total cholesterol, triglycerides, LDL-C, VLDL-C, HDL-C	Bcr/Abl precursor B cells Mouse primary hepatocytes	B cell tumor	Tumor-mediated hyperlipidemia provides LDL cholesterol to support tumor growth.
Khaidakov et al. [61] Ref 60	USA	In vitro	oxLDL-C	Human mammary epithelial cells (MCF10A)	Breast cancer	Mammary epithelial cells respond to oxLDL-C by upregulating proliferative and proinflammatory signals that are part of the oxLDL-C-induced reactions in MCF10A cells. It is mediated by oncogenic hsa-miR-21 by inhibiting its target gene PTEN and triggers the PI3K/Akt pathway.
Llaverias [58] Ref 57	Spain	Mouse model	oxLDL	MMTV-PyMT mice	Breast cancer	oxLDL promotes tumor but supplementation of high-fat diet with phytosterol-enriched diet protects LDL from oxidation.
Lu et al. [35] *Ref 33*	Taiwan	In vitro	HDL-C, LDL-C, and VLDL-C	Cancer cell lines MCF7, HS578T, MDA-MB-468, and MDA-MB-231, and human umbilical vein endothelial cells (HUVEC)	Breast cancer	VLDL-C and LDL-C promote breast cancer cell aggressiveness through enhancing cell migration/invasion and angiogenic activities. However, only VLDL-C provides survival advantage in anchorage-independent condition.
Ma et al. [29] *Ref 27*	China	In vitro In vivo	oxLDL-C	Gastric cancer cell lines HGC-27, MGC-803, and AGS BALB/c nude mice	Gastric cancer	oxLDL promotes expression of VEGF. oxLDL induces NF*κ*B signaling via LOX1. Lymphatic vessel density was significantly higher than adjacent tissues. Weigh and volume of popliteal lymph nodes were significantly increased compared to control.
McCaw [41] Ref 40	Canada	In vitro	LDL-C	Primary chronic lymphocytic leukemia (CLL) cells from human	CLL	LDL degradation product stimulates CLL cells but not the normal lymphocytes.
Moss et al. [44] Ref 43	USA	In vitro In vivo	LDL-C	Primary hepatocytes from Balb/C mice	Liver cancer	Selective LDL-DHA treatment induced toxicity in tumor tissue and cells.
Murtola et al. [40] Ref 38	Finland	In vitro	LDL-C	Prostatic epithelial cell lines (P96E and P97E), in vitro immortalized epithelial cell lines (PWR-1E and RWPE-1), and cancer cell lines (LNCaP and VCaP)	Prostate cancer	Increasing doses of LDL-C induce number of in prostate cancer cells unlike its effect to normal epithelial cells. Both normal and cancer cells increase the production of effectors that ensure the synthesis and uptake of cholesterol under depletion.
Navarro et al. [42] Ref 41	Spain	In vitro	Total cholesterol Triglycerides	Rat hepatoma cells	Hepatocellular carcinoma cells	SND1 oncoprotein induces cholesterol ester accumulation. It causes lower use of fatty acid for triglyceride synthesis.
Ou et al. [45] Ref 44	USA	In vitro		Human liver tumor cell lines (PLC/PRF/5) Rat hepatoma cell line (H4HE)	Hepatocellular carcinoma	LDL-DHA induces cell death in hepatocellular carcinoma via ferroptosis pathway.
Rodrigues dos Santos et al. [25, 26] Ref 39	Portugal	In vitro	LDL-C	Cancer cell line HTB20, MDA MB 231	Breast cancer	Microarray analysis shows overexpression of Akt and ERK pathway intermediates suggesting LDL-C induces survival response.
Rodrigues et al. [42] Ref 34	Portugal	In vitro In vivo	LDL-C	Vg9Vd2 T cells, MDA-MB-231 Xenograft mouse model	Breast cancer	LDL-C causes impaired IFN*γ* and lower cytotoxicity by T lymphocyte.
Scoles et al. [57] Ref 56	USA	In vitro	oxLDL-C	Ovarian carcinoma cells CAOV3, ES2, OVCAR3, PA1, SKOV3, SCOC882, CSOC909, A2780, and CP70	Ovarian cancer	oxLDL in low concentration causes stimulation of ovarian cancer cell than LDL.
Sobot et al. [37] Ref 35	Germany	In vitro	LDL-C	MDA-MB-231. MCF7, CK-OV-3, A549	Breast cancer Ovarian adenocarcinoma Adenocarcinoma alveolar basal epithelial	Presence of more lipid droplets in MDA-MB-231 cells that express higher LDL receptor (LDLR)
Wan et al. [23] Ref 22	China	In vitro	oxLDL-C	Cancer cell lines, LNCaP and PC-3	Prostate cancer	oxLDL-C stimulates proliferation, migration, and invasion of LNCaP and PC-3.
Wang et al. [60] Ref 59	China	In vitro	oxLDL-C	Human lung cancer cells HCC827, A549, H441, H446, H460, and H522	Lung adenocarcinoma	Increase uptake of oxLDL promotes metastasis via C/EBP6 regulation.
Wen et al. [43] Ref 42	United States	Animal model	LDL	Rat	Hepatocellular carcinoma	Hepatocellular carcinoma cells exhibit uptake of LDL nanoparticles. Incorporation of HAD provides selective targeting of cancer cells.
Zhao et al. [39] Ref 37	China	In vitro Human tissue	Total cholesterol Triglycerides	Hepatoma cell lines (HepG2, H7402, Huh7, MHCC-97L, MHCC-97H, and SMCC-7721) Hepatocellular tissues	Hepatocellular carcinoma	Accumulation of triglycerides, total cholesterol, and lipid droplet is stimulated by SPIN1 via FASN.

## Data Availability

The data supporting this systematic review are from previously reported studies, which have been cited. The data are available from the corresponding author upon request.

## Abbreviations

ABCA1: ATP-binding cassette transporter
Apo A-I: Apolipoprotein A1
Apo A-II: Apolipoprotein A-II
Apo A-IV: Apolipoprotein A-1V
Apo C: Apolipoprotein C
Apo E: Apolipoprotein E
BC: Breast cancer
BMI: Body mass index
CI: Confidence interval
CM: Chylomicrons
CRC: Colorectal cancer
EM: Eye metastasis
ER+BC: Estrogen receptor-positive breast cancer
ER-/PR-: Estrogen receptor negative/progesterone receptor negative
HDL: High-density lipoprotein
HDL-C: High-density lipoprotein cholesterol
HMG-CoA: 3-Hydroxy-3-methyl-glutaryl-CoA
HR: Hazard ratio
LCFA: Long chain fatty acids
LDL: Low-density lipoprotein
LDL-C: Low-density lipoprotein cholesterol
LPL: Lipoprotein lipase
NPC: Nasopharyngeal carcinoma
OLR1: Oxidized low-density lipoprotein receptor 1
OR: Odds ratio
oxLDL: Oxidized low-density lipoprotein
PCa: Prostate cancer
TC: Total cholesterol
TG: Triglycerides
VLDL: Very low-density lipoprotein
VLDL-C: Very low-density lipoprotein cholesterol.
